# Flexibility of Physiological Traits Underlying Inter-Individual Growth Differences in Intertidal and Subtidal Mussels *Mytilusgalloprovincialis*

**DOI:** 10.1371/journal.pone.0148245

**Published:** 2016-02-05

**Authors:** María José Fernández-Reiriz, Jade Irisarri, Uxio Labarta

**Affiliations:** Consejo Superior de Investigaciones Científicas (CSIC), Instituto de Investigaciones Marinas (IIM), Vigo, Spain; University of Siena, ITALY

## Abstract

Mussel seed (*Mytilusgalloprovincialis*) gathered from the intertidal and subtidal environments of a Galician embayment (NW, Spain) were maintained in the laboratory during five months to select fast (F) and slow (S) growing mussels. The physiological basis underlying inter-individual growth variations were compared for F and S mussels from both origins. Fast growing seemed to be a consequence of greater energy intake (20% higher clearance and ingestion rate) and higher food absorption rate coupled with low metabolic costs. The enhanced energy absorption (around 65% higher) resulted in 3 times higher Scope for Growth in F mussels (20.5±4.9 J h^−1^) than S individuals (7.3±1.1 J h^−1^). The higher clearance rate of F mussels appears to be linked with larger gill filtration surface compared to S mussels. Intertidal mussels showed higher food acquisition and absorption per mg of organic weight (i.e. mass-specific standardization) than subtidal mussels under the optimal feeding conditions of the laboratory. However, the enhanced feeding and digestive rates were not enough to compensate for the initial differences in tissue weight between mussels of similar shell length collected from the intertidal and subtidal environments. At the end of the experiment, subtidal individuals had higher gill efficiency, which probably lead to higher total feeding and absorption rates relative to intertidal individuals.

## Introduction

The existence of high inter-individual variability in growth is a major problem for aquaculture and positions bivalves as prime candidates for size-based selective breeding programs to increase the production of shellfish farms. The physiological traits involved in growth performance are modulated through a combination of endogenous and exogenous (food environment) factors. Bayne (1999) [1] proposed that inter-individual growth differences among individuals living under similar environmental conditions may be achieved by: a) faster energy acquisition through increased food consumption and/or absorption; b) more efficient allocation of energy for maintenance, growth and reproduction, and c) lower metabolic costs of growth. Bivalves would attain higher Scope for Growth (i.e. energy available for growth and reproduction) through the combination or single utilization of the former strategies. Bayne (1999) [1] found that faster growth was supported by higher energy acquisition (i.e. higher clearance rate, CR and ingestion rate, IR) and lower oxygen consumption and ammonia excretion in two genetically distinct lines of oysters (*Crassostreagigas*) selected for fast growth compared to control oysters. Recent studies have further tested the physiological basis underlying the endogenous inter-individual differences in growth in clams *Ruditapesphilippinarum* [2, 3, 4] and oysters *Crassostreagigas* [5]. These studies were in agreement with the hypothesis proposed by Bayne (1999) [1] and linked persistent inter-individual differences in growth rates with endogenous genetic-based differences in the rates of food acquisition, absorption and metabolic costs.

The inter-individual variation in bivalve growth is of major importance for regions that sustained large aquaculture productivity like the region of Galicia (NW Spain), where mussel (*Mytilusgalloprovincialis*) seed supports a production of 250,000 tonnes per year. The raft culture process requires large amount of mussel seed that is attached to ropes that will be thinned out twice before reaching commercial size [6,7]. Mussel seed can be obtained from two different origins, either from direct harvest in exposed rocky shores (i.e. intertidal environment) or from artificial collector ropes hung from mussel rafts during the major spawning events in spring and autumn (i.e. subtidal environment) [6,7]. However, since Galician coastal management plansrestrict the number of collector ropes per raft (maximum100 collector ropes of 12 m), most of the seed is obtained from the rocky shore, with the exception of experimental licenses authorized to certain rafts and long-lines in Ría Ares-Betanzos. The pioneering study of Pérez-Camacho et al. (1995) [8] investigated the influence of the origin of the seed stock upon subsequent mussel growth after cultivating intertidal and subtidal seed in rafts. In this study, Pérez-Camacho et al. (1995) [8] observed a higher growth rate in mussel seed from collector ropes and concluded that the utilization of seed with subtidal origin could reduce the total duration of the mussel culture by more than 10%. Subsequent studies furtheranalyzed the endogenous differences in physiological energetics [9,10,11], biochemical composition [12, 13, 14] and growth rate [15] between mussel seed (*Mytilusgalloprovincialis*) gathered from the intertidal environment with respect to those gathered from the subtidal environment in the Galician Rías (NW Spain). The outcome of these studies revealed that bivalves exhibit inter-individual variability in growth rates and physiological energetics when reared under the same environmental conditions [8, 9, 10, 11]. Seed originated from artificial collectors is previously adapted to subtidal conditions, which probably explains the higher amount of glycogen and lipid reserves compared to rocky shore seed [14]. Furthermore, Labarta et al. (1997) [9] measured higher Scope for Growth (SFG) in raft-cultured mussels with respect to intertidal mussels after 15 d of maintenance in standard laboratory conditions, mainly due to the higher clearance rate (CR) and absorption efficiency (AE) of subtidal mussels.

This study analyzed the physiological basis underlying inter-individual growth variations in mussel (*Mytliusgalloprovincialis*) seed from two distinct origins, the intertidal and subtidal environments. Individuals from both origins had the sameinitial size and were reared during five months in the laboratory to select individuals with the fastest (F) and slowest (S) growth rates. The aim of the experiment was to identify the physiological components of the energy balance equation (i.e. Scope for Growth) that explain the differences in growth between fast and slow growing mussels. A second objective was to compare the Scope for Growth between F and S mussels from the subtidal and intertidal environments.

## Materials and Methods

### 2.1. Experimental design

Mussel (*Mytilusgalloprovincialis*) seeds were obtained from two different origins in the Ría Ares-Betanzos (NW Spain) in September 2014: artificial rope collectors suspended from a commercial mussel raft (subtidal environment) and the rocky shore (intertidal environment). Mussel seed from both sites belonged to the same massive spawning event that typically occurs in the Galician Rías in spring. Mussels were transported to the laboratory and segregated in three groups based on their average shell length: 19 mm, 20 mm and 21 mm. The shell length of each individual was recorded to the nearest 0.1 mm with callipers. Mussels from the subtidal had an average initial dry weight (DW) of the tissues of 47.01±7.16 mg, a DW of the shell of 255.5±35.8 mg and an ash content of 15.81±0.60% (n = 90, 30 individuals per size class). Mussels from the intertidal had an average DW of the tissues of 38.10±6.77 mg, a DW of the shell of404.8±78.2 mg and an ash content of 17.89±1.60% (n = 90, 30 individuals per size class). Bivalves were placed in 6 flow-through plastic aquarium (44.5x40x14.5 cm, 19 l seawater volume). Each aquarium contained 400 mussel seeds. Mussels were maintained during five months in each aquarium with a continuous flow of 1200 ml min^−1^ of seawater at 15°C and 35‰ salinity. The diet during the maintenance period consisted of a monoalgal suspension of *Rhodomonas lens* supplied in a continuous flow to each aquarium by a peristaltic pump (ISMATEC MPC Process) to maintain a concentration of 10,000 cells ml^−1^ with 99% organic content. The diet during the physiological experiments consisted of a mixture of *R*. *lens* and freeze-dried sediment (proportion 40:60 w:w). The use of sediment in the diet aimed to fulfill the requirement of an inorganic tracer during the measurements of the absorption efficiencyaccording Conover (1966) [16]. Mussels were acclimated to this mixture of algae and sediment during 7 days prior to the start of the physiological experiments. The diets were maintained in an aerated tank of 60 l to generate a homogeneous mixture and prevent sedimentation. *R*. *lens*maximizes the feeding, digestion and the assimilatory balance of nutrients and energy in mussel *M*. *galloprovincialis* compared to other classical monoalgal diets [17]. Mortality during this prolonged maintenance period was less than 5%. After 5 months, individuals that represented the extreme percentiles of the size-distribution (P-15 and P-85) were selected to obtain the fastest (F: 36 to 39 mm shell length) and slowest (S: 20 to 23 mm shell length) growers from the intertidal and subtidal environments. Physiological determinations of feeding, digestion, respiration and excretion were performedat time 0 and after 5 months of maintenance in the lab. Physiological parameters were determined using 20 samples for each size class (F and S) and origin (subtidal and intertidal). Samples in each group were intended to have the same live biomass and thus, comprised 3 individuals in F group and 6 individuals in S group. Individuals in each replicate were randomly selected. Each measurement obtained for the absorption efficiency was the result of pooling the fecal material of 2 to 3 samples, in order to obtain enough dry weigh to determine the organic and inorganic content of the feces. In addition, 30 individuals per group were dissected for tissue and shell DW determinations (100°C, 24h). The bulk tissue was then dried at 450°C during 24 h for ash-content determinations. The Condition Index (CI) was calculated as: (DWtissue/ DWshell) x 100. The growth rates of the tissue and shell were calculated as: (DWfinal-DWinitial)/t.

### 2.2. Diet characteristics

Diet samples were taken in triplicate to determine the particulate characteristics of the diet used during the physiological experiments. Samples were filtered onto Whatman GF/C fiberglass filters that had been previously washed, ashed and weighed. Filters were rinsed with a solution ofammonium formate0.5 M to remove sea salt. Total particulate matter (TPM, mg l^−1^) was obtained after oven-drying the filters at 100°C until constant weight. Filters were then ashed at 450°C in a muffle furnace to determine the inorganic matter (PIM, mg l^−1^). The particulate organic matter(POM, mg l^−1^) was calculated by difference between the TPM and the PIM. The organic fraction (*f*) was computed as POM/TPM. The diet had the following characteristics: 0.72±0.06 mg l^−1^ TPM, 0.59±0.03 mg l^−1^ POM, 18.96±4.79% ash, 0.81±0.05 *f*.

### 2.3. Physiological determinations

The clearance rate (CR, ml h^−1^) was estimated as the reduction in suspended particle concentration between water surrounding the individuals and the outflow of the experimental chamber as described in Filgueira et al. (2006) [18]. The organic ingestion rate (OIR, μgh^−1^) was calculated as the product of the CR x POM. The absorption efficiency (AE, %) was estimated by determining the organic and inorganic content of the diet and the mussels’ feces following the method of Conover (1966) [16]. The absorption rate (AR, mg h^−1^) was calculated as the product of the AE x OIR. For respiration rate (VO_2_, μlO_2_ h^−1^) determinations, mussels were incubated in sealed chambers with seawater until O_2_ concentrations fell below 30% of the initial value. Two chambers without bivalves were used as a control. The decline in O_2_ concentration was monitored with YSI58 oxygen meters connected to YSI5730 probes. The VO_2_ was determined as the difference in O_2_ concentration between the experimental and control chambers. The ammonia excretion rate (VNH_4_-N, μg NH_4_-N h^−1^) was obtained after incubating the mussels in chambers with filtered seawater. Two empty chambers were used as a control. TheNH_4_-N present in the water was determined with the phenol-hypochlorite method (Solorzano, 1969) [19]. VNH_4_-N was calculated as the difference in ammonia concentration between the experimental and control chambers. The ratio of oxygen consumed to nitrogen excreted (O:N) was computed by atomic equivalents, according to Widdows (1985) [20]. A detailed description of the physiological methods can be found in Fernández-Reiriz et al. (2012) [21].

The Scope for Growth (SFG, Jh^−1^) is the fraction of the absorbed energy available for somatic and gametogenic growth once metabolic requirements were met (Widdows, 1985) [20]. The SFG was computed with the following energy balance equation (Winberg, 1960; Ivlev, 1966) [22,23]:

SFG=I−Fae−M=AR−M

Where I is the ingested energy, Fae is the energy loss in the faeces, M summarizes the metabolic expenditure plus the energy loss due to excretion and AR is the absorbed energy (IR x AE). The following energy conversion factors were used (Bayne et al., 1985) [24]:

1 mg POM = 23.5 J

$$1\;ml\;O_{2}=20.36\;J$$

$$1\mu g\;NH_{4}N=0.0249\;J$$

Physiological rates were expressed as mass-specific rates (Y_SPC_) in mg of organic weight (OW).

### 2.4. Gill area and gill efficiency

The gill area (mm^2^) was estimated fitting the shell length of each mussel to the equation proposed by Filgueira et al. (2008) [25] that demonstrated that the relationship between gill area and shell length remains constant and independent of the condition index:

$$Gill\;area=1.48\;x\;Length^{1.93}\;\left(R^{2}=0.99\right)$$

Gill area was estimated from 15–20 individuals previously used for the physiological measurements for each size class (F and S) and origin (subtidal and intertidal). The gill efficiency (ml h^−1^ mm^-2^) was calculated as the ratio of the CR to the gill area.

### 2.5. Statistical analyses

The significant effect exerted by the origin of the mussel seed (i.e. subtidal *vs* intertidal environment), the growth group (i.e. fast ‘F’ *vs* slow ‘S’ growers) and the interaction of both factors on the different physiological components of the Scope for Growth was analyzed by two-way ANOVA followed by Tukey’s HSD as a post-hoc test. The DW of the tissue and the shell, ash content, growth ratesand gill efficiency were also compared by means of two-way ANOVA. Normality and homogeneity of variances were verified prior to performing ANOVA with Shapiro-Wilk and Levene test, respectively. Whenever these assumptions were violated we performed ANOVA on ranks after the ranked transformation of the data. Statistical analyses were performed using Statistica 7.0. (StatSoft, Inc.).

## Results

### 3.1. Ash content, dry weight, Condition Index and growth rates

The initial biometric characteristics determined for the juvenile mussels are shown in Table 1.

**Table 1:** Biometric parameters (mean ± SD) of mussel (*Mytilusgalloprovincialis*) seed from subtidal and intertidal origins obtained at the beginning and after 5 months acclimation in the laboratory to select the individuals with slow (S) and fast (F) growth rates.

	Initialvalues		Acclimationlaboratory			
	Subtidal	Intertidal	Subtidal		Intertidal	
			S	F	S	F
Length (mm)	19–21	19–21	20–23	36–39	20–23	36–39
Tissue DW(mg)	47.0±7.1	38.1±6.7	79.2±4.6	187.0±23.3	59.6±4.3	135.3±26.8
Shell DW (mg)	255.5±35.8	404.8±78.2	535.0±41.0	1482.2±118.2	554.8±57.5	1139.6±122.1
Shell DW / Meat DW	5.4	10.6	6.7	7.9	9.3	8.4
Tissue growth rate	-	-	6.4	28.0	4.3	19.4
Shell growth rate	-	-	55.9	245.3	29.9	146.9
Ash (%)	15.8±0.6	17.8±1.6	20.8±2.7	24.7±0.3	14.1±2.6	21.6±1.9

Values are given as dry weight (DW). The growth rates (mg month^−1^) were calculated as: (DW_final_−DW_initial_)/t.

**Fig 1:**
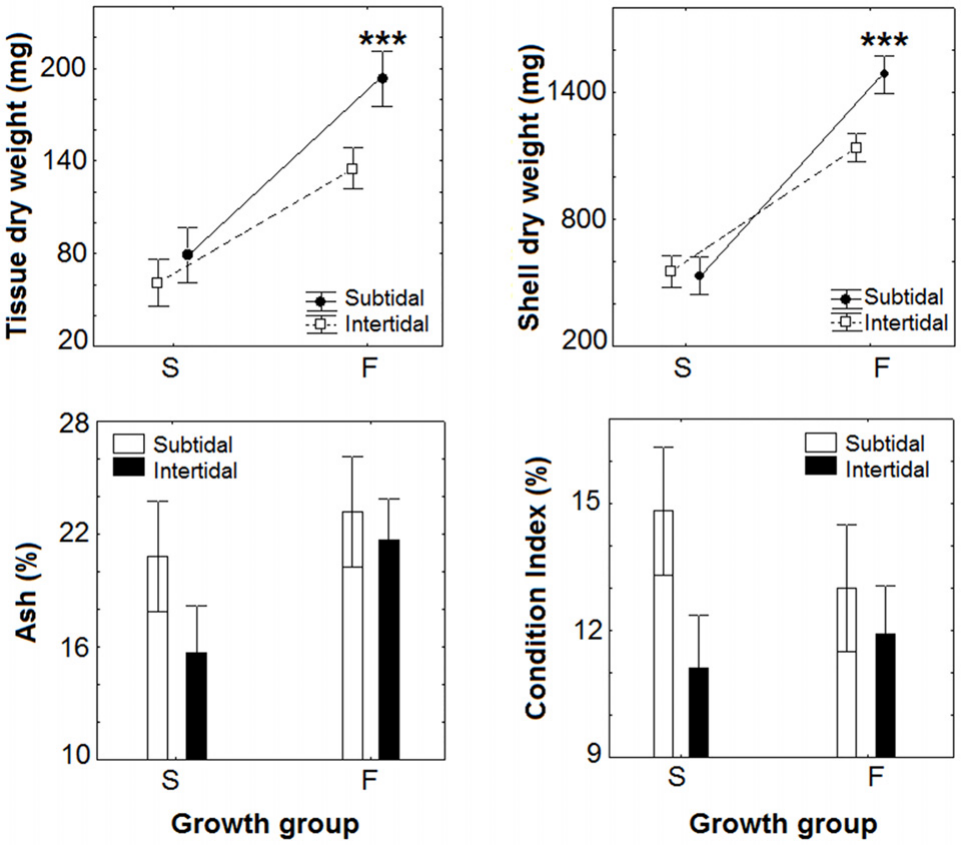
Biometric characteristics of slow (S) and fast (F) growing mussel seed originated from the subtidal and intertidal ecosystems after maintenance under standard laboratory conditions during five months. The graphs represent the mean ± SD of the tissue and shell dry weight, the percentage of ash and the condition index. Statistical differences are indicated as *** for p<0.001 (two-way ANOVA).

The two-way ANOVA showed that the ash content, the DW of the tissue and shell varied significantly with the growth group, origin and the interaction term (group x origin), excepting for a lack of significance of the interaction term on the ash content (S1 Table). Thus, fast-growing individuals (F) showed significantly higher levels of ash and DW of the tissue and shell compared to slow-growing (S) mussels (Tukey’s test, p<0.05; Fig 1). Subtidal mussels showed greater content of ash, DW of the tissue and shell than intertidal bivalves (Tukey’s test, p<0.05; Fig 1). Fast-growing subtidal mussels had higher DW of the tissue and shell than F intertidal mussels (Tukey’s test, p<0.05; Fig 1).

The Condition Index only varied significantly depending on the origin, with higher values in subtidal mussel seed (13.9±1.2%) than in intertidal mussels (11.5±1.9%) (two-way ANOVA, p<0.001; S1 Table and Fig 1). Two-way ANOVA pointed that the growth rates for the tissue and the shell varied significantly with the growth group, origin and the interaction term (group x origin) (F_(1,30)_ = 20.9, p<0.001 and F_(1,30)_ = 43.9, p<0.001, respectively). The growth rate of the tissue and the shell was significantly higher in F growers (Tukey’s test, p<0.05; Table 1). Subtidal mussels showed comparatively greater growth rates than intertidal individuals (Tukey’s test, p<0.05; Table 1). Lastly, F growers from the subtidal had significantly higher growth rates than intertidal individuals (Tukey’s test, p<0.05; Table 1).

### 3.2. Feeding components of the energy balance

The feeding rates determined for mussel seed at the beginning of the experiment and after five months of maintenance in the laboratory are shown in Table 2.

**Table 2:** Physiological parameters (mean ± SD) of mussel (*Mytilusgalloprovincialis*) seed from subtidal and intertidal origins. The table shows the initial values of the energy balance and the physiological parameters obtained after 5 months of maintenance the laboratory to select the individuals with slow (S) and fast (F) growth rates. The physiological measurements included the clearance rate (CR; OW:organic weight), ingestion rate (IR), absorption efficiency (AE) and absorption rate (AR), respiration rate (VO_2_), ammonia excretion rate (VNH_4_-N) and Scope for Growth (SFG). Physiological rates were expressed as mass-specific rates (Y_SPC_) in mg of organic weight (OW).

	Initial values		Acclimation laboratory			
	Subtidal	Intertidal	Subtidal		Intertidal	
			S	F	S	F
CR _(total)_ (ml h^−1^)	554.4±50.5	549.0±24.9	682.5±75.9	1865.0±268.7	636.7±97.1	1589.7±408.1
CR_(SPC)_ (ml(mgOW)^−1^h^−1^)	13.9±1.2	17.5±0.8	10.8±1.2	12.8±1.8	12.2±1.7	14.9±3.8
OIR _(total)_ (μg OW h^−1^)	396.4±36.1	392.6±17.8	443.0±49.3	1335.3±192.4	466.7±71.1	1211.4±310.9
OIR _(SPC)_ (μg OW (mgOW)^−1^ h^−1^)	10.0±0.91	12.5±0.5	7.0±0.7	9.2±1.3	9.1±1.2	11.4±2.9
AE (%)	89.1±0.7	86.6±0.6	80.6±0.5	80.8±3.5	78.8±5.5	81.5±3.8
AR _(total)_ (mg OW h^−1^)	0.3±0.09	0.3±0.01	0.3±0.03	1.7±0.1	0.3±0.05	0.9±0.2
AR _(SPC)_ (μg OW (mgOW)^−1^ h^−1^)	8.9±0.8	10.9±0.5	5.7±0.6	7.4±1.1	7.2±1.1	9.3±2.4
VO_2 (total)_ (μlO_2_ h^−1^)	48.0±11.5	46.0±5.5	60.8±11.6	147.1±19.4	65.4±9.6	148.6±17.8
VO_2 (SPC)_ (μlO_2_ (mgOW)^−1^h^−1^)	1.3±0.4	1.7±0.4	0.9±0.2	1.0±0.1	1.2±0.19	1.2±0.2
VNH_4_-N _(total)_ (μgNH_4_-N h^−1^)	2.2±0.7	1.9±0.2	2.0±0.6	5.6±0.6	1.71±0.6	5.6±1.7
VNH_4_-N_(SPC)_(μgNH_4_(mgOW)^−1^h^−1^)	0.05±0.01	0.06±0.01	0.03±0.01	0.04±0.01	0.03±0.01	0.05±0.02
O:N index	29.7±11.7	30.0±3.9	41.8±18.1	32.6±6.1	52.7±17.4	37.7±12.6
SFG (J h^−1^)	8.2±0.7	7.8±0.4	7.5±0.9	21.7±3.3	7.3±1.3	19.8±2.0

**Fig 2:**
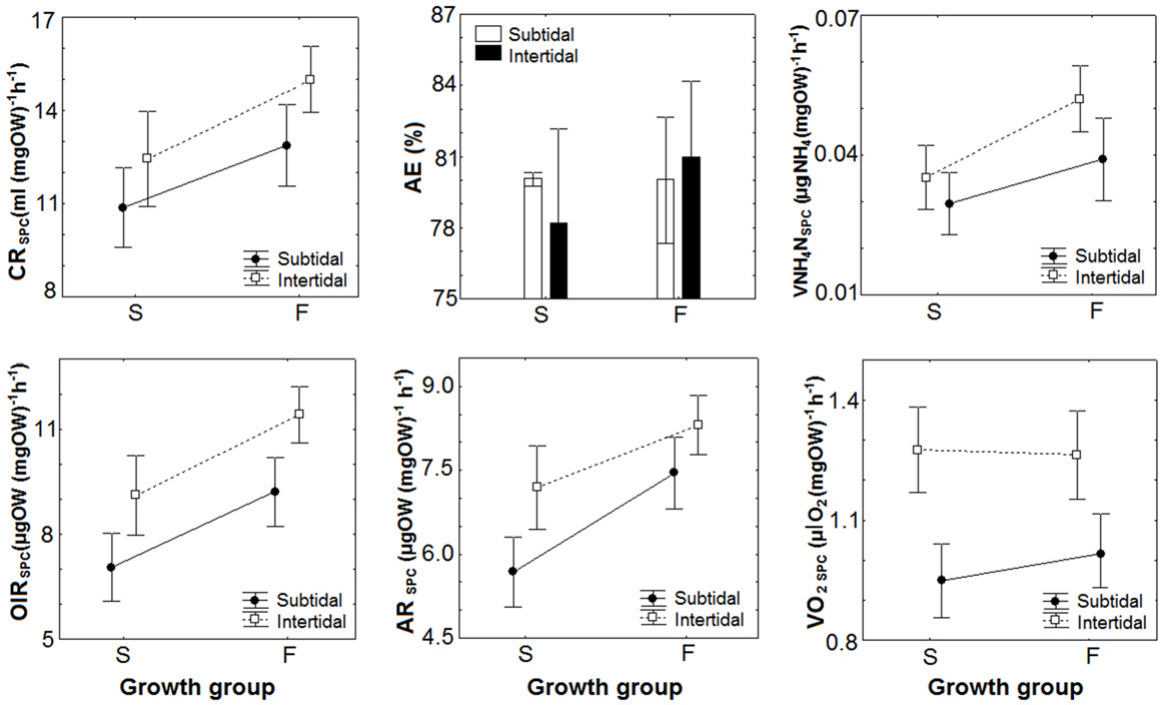
Mass-specific physiological components of the energy balance in slow (S) and fast (F) growing mussel seed originated from the subtidal and intertidal ecosystems and maintained under standard laboratory conditions during five months. The feeding (clearance rate: CR, and ingestion rate: IR), digestive (absorption efficiency: AE, and absorption rate: AR) and metabolic parameters (ammonia excretion: VNH_4_-N and, oxygen consumption: VO_2_) represent the mean ± SD.

Two-way ANOVA showed a significant effect of the growth group on the mass-specific feeding rates (S2 Table). At the end of the experiment, themass-specific CR and OIR were significantly higher in F mussels than in S mussels (Tukey’s test, p<0.05; Fig 2). Likewise, the total CR (F_(1.65)_ = 235.50, p<0.001) and total OIR (F_(1.65)_ = 246.02, p<0.001) were higher in F individuals (two-way ANOVA). The mass-specific feeding rates varied significantly with the origin of the mussel seed (two-way ANOVA, p<0.05; S2 Table). Intertidal individuals showed higher mass-specific feeding rates than subtidal mussels (Tukey’s test, p<0.05; Fig 2). However, the two-way ANOVA indicated that subtidal individuals had the highest total CR (F_(1.65)_ = 5.32, p<0.05) and OIR (F_(1.65)_ = 0.92, p<0.05).

We found no significant effect of the interaction term (origin x growth group) on the feeding rates (two-way ANOVA, p>0.05; S2 Table).

### 3.3. Digestive components of the energy balance

The absorption efficiency was not significantly different between F and S mussels or between subtidal and intertidal individuals (mean 80.4±4.3% AE) (two-way ANOVA, p>0.05; S2 Table, Fig 2).

Two-way ANOVA showed that the AR per unit mass varied significantly with the growth group and the origin of the mussels, but there was no significant effect of the interaction term (group x origin) (S2 Table). The AR_(SPC)_ was greater in F mussels than in S mussels (Tukey’s test, p<0.05; Fig 2). Mussels from the intertidal showed higher AR_(SPC)_ than those from the subtidal origin (Tukey’s test, p<0.05; Fig 2). F mussels also showed a greater total AR than S mussels (two-way ANOVA, F_(1, 65)_ = 301.29, p<0.001) but subtidal individuals had a higher total AR (two-way ANOVA, F_(1, 65)_ = 3.97, p<0.05).

### 3.4. Metabolic components of the energy balance

The VO_2 (SPC)_only varied significantly with the origin (two-way ANOVA, p<0.001; S2 Table). The highest VO_2_ wasregistered in intertidal individuals relative to subtidal mussels (Tukey’s test, p<0.05; Fig 2). The same pattern was identified for the VO_2(total)_ (two-way ANOVA, F_(1,65)_ = 0.43, p<0.05).

Two-way ANOVA showed that the VNH_4_-N_(SPC)_ varied significantly with the growth group and the origin of the mussels, but there was no significant effect of the interaction term (group x origin) (S2 Table). F mussels excreted more ammonia than S individuals and intertidal mussel seed produced more ammonia than subtidal mussels (Tukey’s test, p<0.05; Fig 2). The total VNH_4_-N was also greater in F mussels (F_(1,65)_ = 150, p<0.001), although it was similar for bivalves from both origins (F_(1,65)_ = 0.74, p>0.05).

The O:N index was significantly greater in S mussels than in F individuals (two-way ANOVA, F_(1,55)_ = 8.66, p<0.001), although it did not vary depending on the origin (two-way ANOVA, F_(1,55)_ = 3.73, p>0.05; Table 2).

### 3.5. Scope for Growth

**Fig 3:**
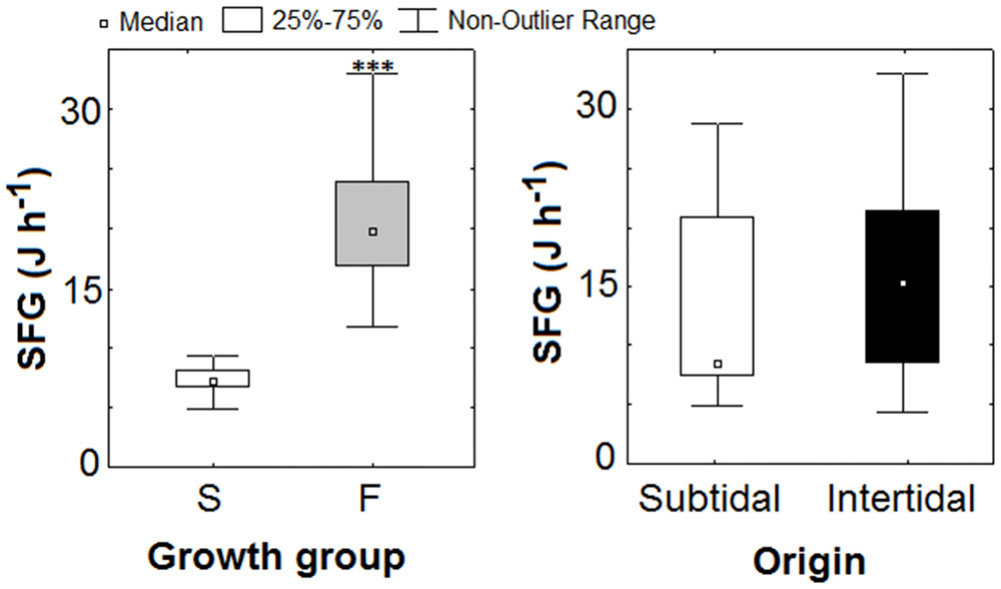
Box-plots comparing the Scope for Growth (SFG, J h^−1^) of slow (S) and fast (F) growing mussel seed (left panel) and the Scope for Growth of subtidal (mussel raft) and intertidal (rocky shore) mussel seed gathered from the Ría Ares-Betanzos (NW, Spain). Statistical differences are indicated as *** for p<0.001 (two-way ANOVA).

The Scope for Growth only varied significantly depending on the growth group of the mussels (two-way ANOVA, p<0.001; S2 Table), as F mussels had higher SFG (mean 20.5±4.9 J h^−1^) than S mussels (mean 7.3±1.1 J h^−1^) (Tukey’s test, p<0.001; Fig 3). Intertidal and subtidal individuals showed similar values (mean 15.0±7.6 J h^−1^) (two-way ANOVA, p>0.05; Fig 3).

**Fig 4:**
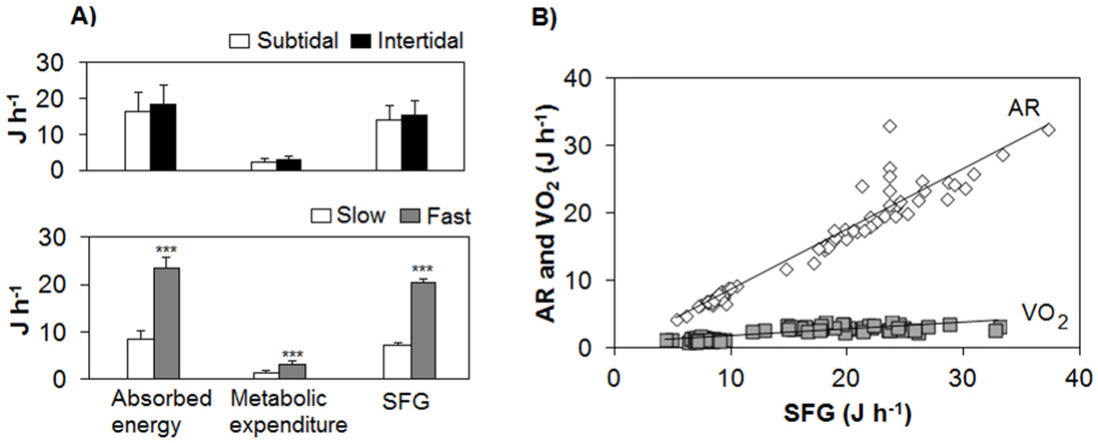
A) Comparison of the absorbed energy (AR = IR x AE), metabolic energy expenditure (oxygen consumption and ammonia excretion, M) and SFG of the two origins and growth groups of *M*. *galloprovincialis*. Statistical differences are indicated as *** for p<0.001 (two-way ANOVA). B) Absorption rate (AR: diamonds) and oxygen consumption (VO_2_: squares) at the different levels of Scope for Growth (SFG) obtained for intertidal and subtidal individuals after five months of maintenance with a monoalgal diet of *Rhodomonas lens*. Equations of regression lines fitted by least-squares to all the combined data are: AR = 0.89 SFG– 0.25 (R^2^ = 0.92) and VO_2_ = 0.10 SFG + 0.74 (R^2^ = 0.67).

This was a consequence of the significantly higher AR and M of fast-growing mussels compared to slow-growing individuals (two-way ANOVA, F_(1,65)_ = 251.26 and F_(1,65)_ = 504.18, p<0.001), although the higher M in F growers was due to the fact that they excreted more ammoniathan S growers (Fig 4). AR and M did not vary with the origin of the mussels (Fig 4; two-way ANOVA, p>0.05).

### 3.6. Gill area and gill efficiency

**Fig 5:**
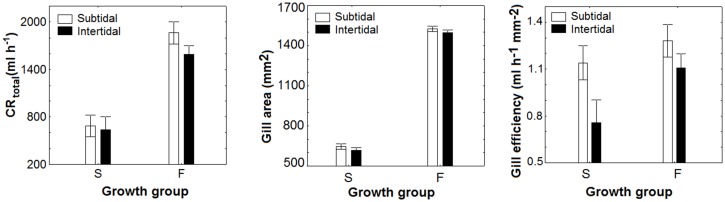
Comparison of the total clearance rate (CR_total_), gill area and gill efficiency (ratio of the CR_total_ to the gill area) obtained in slow (S) and fast (F) growing mussel seed originated from the subtidal and intertidal ecosystems after maintenance with a monoalgal diet of *Rhodomonas lens* during five months.

The gill area reflected the differences in size between Fand S growers in agreement with the allometric equation proposed by Filgueira et al. [25] (Fig 5).

The gill efficiency was significantly different depending on the growth group and the origin of the mussels. A significant effect of the interaction term (group x origin) was not observed (two-way ANOVA, p>0.05; S1 Table). The gill efficiency was significantly higher in subtidal mussels than in intertidal individuals (Tukey’s test, p<0.05; Fig 5). The gill efficiency was higher for S mussels (0.86 ±0.11 ml h^−1^ mm^-2^) than F mussels (0.78 ±0.17 ml h^−1^ mm^-2^) (Tukey’s test, p<0.05; S1 Table; Fig 5).

## Discussion

### 4.1. Origin-related physiological differences

The initial physiological parameters were comparable to that previously reported for subtidal and intertidal mussels (Labarta et al. 1997) [9]. The comparison of the physiological responses of mussels from two different coastal habitats revealed that intertidal mussels had a higher CR and IR per unit mass, which was associated with faster AR_(SPC)_ of nutrients across the digestive tract and eventually resulted in a slight increase of the metabolic expenditure compared to subtidal individuals. Babarro et al. [10, 11] also evaluated the feeding behavior and metabolism in mussel seed from the rocky shore (intertidal) and collector ropes (subtidal) after cultivating mussels from both origins on a raft from a length of 20 to 60 mm during 226 d. Intertidal mussels increased the CR and OIR during day 22 up to day 110 of culture, which lead to a higher VO_2_. The intertidal mussels also showed a higher VNH_4_-N and a lower O:N index, which may indicate a slow process of adaptation owing to osmotic adjustments in which the pool of free amino acids plays an important role [10]. The fact that feeding and digestive rates of intertidal individuals surpass that of subtidal mussels suggests the need of short-term physiological adaptations to the new environmental conditions. Mussels living in the intertidal zone have access to an intermittent food source, since they cease feeding when exposed to air during the low tide. In contrast, mussels cultured in rafts in the subtidal zone are previously adapted to continuous immersion and have access to a constant supply of food (seston) of more stable quality and quantity. Thus, it is probable that the initial physiological measurements reflected the need of a comparatively higher feeding and digestive activity per unit mass to match the intermittent food supply found in the intertidal, leading to a similar SFG in mussels from both origins. On the other hand, after five months of maintenance in the laboratory, bivalves from both origins continued to have a similar SFG, probably owing to the higher feeding and digestive rates per unit mass registered in intertidal mussels as a response to the continuous food supply. The results of our study were in line with those of Labarta et al. [9], who compared the physiological energetics of subtidal and intertidal mussels after 15 d of exposure to experimental diets. Intertidal mussels responded better to the improved feeding conditions in the laboratory and increased the SFG by 39% compared to initial values, while subtidal mussels only incremented the SFG by 20%. Nonetheless, subtidal mussels presented an overall higher weight-specific CR and AE, which resulted in an increased SFG compared to intertidal mussels [9]. The results of our study showed that the higher feeding and digestive rates of intertidal mussels were not enough to compensate for the initial differences in tissue weight between subtidal and intertidal mussels of similar shell length (47 and 38 mg initial DW tissue, respectively). Even though mussels from both origins reached an equal length at the end of the experiment they still differed in weight, with 30% higher tissue weight in subtidal mussels. Similarly, in the experiment of Tedengren et al. [26], the transplanted population of *Mytilusedulis* from the Baltic Sea reached the same length than native mussels from the North Sea, but showed a lower tissue weight even after one year of being transplanted. Other experiments showed that origin-related differences in the CR, AE and Condition Index of mussels persisted after 8 weeks of being transplanted to three different cultivation rafts with similar ambient conditions [27]. Thus, even if intertidal mussels clearedmore particles permg OW, it is important to note that subtidal individuals had greater tissue weight, which would lead to a higher total food capture area relative to intertidal individuals. A larger gill area was also accompanied by significantly higher CR when comparing different populations of an infaunal bivalve [28] and when comparing two species of bivalves from the intertidal and infaunal environments [29]. The enhanced gill efficiency might be a morphological adaption to the feeding environment [28, 30]. Intertidal mussels face rapid fluctuations in food quality and quantity during the tidal cycle and are also exposed directly to wave-induce sediment resuspension compared to mussels living in the subtidal environment. A reduction in gill size could prevent saturation of the feeding organs in environments with a high influx of sediments [30]. Intertidal mussels, however, showed a heavier shell than subtidal individuals at the beginning of the experiment and after 5 months of maintenance in the lab (Table 1). Origin related differences for subtidal and intertidal mussels also revealed a different allocation of energy for shell (61% and 73% of total energy, respectively) and tissue production (39% and 27% of total energy, respectively) at the beginning of the experiment (see S3 Table based on energetic equivalents by Wolowicz andGoulletquer, 1999) [31]. These differences persisted after 5 months, as intertidal mussels allocated more energy for the production of a thicker shell than subtidal mussels. In fact, the growth efficiency (i.e. rate of growth as a proportion of absorption rate) of subtidal mussels was greater than that of intertidal individuals (S3 Table). This might be interpreted as a physiological strategy to cope with environmental changes in the intertidal environment like wave-derived mechanical stress, desiccation during ebb tide or protection against predators. The greater proportion of the total energy allocated for shell growth in intertidal bivalves provides new evidence of the inter-individual physiological differences in growth initially suggested by Bayne [1]. Early papers suggested that the process of shell formation demands around 33% of the total energy used for growth [32]. Our experimental results highlight that shell growth represents a great energetic cost for bivalves and that the amount of energy allocated for shell growth varies depending on the environment.

The fact that physiological and biometric differences associated with the origin are persistent in transplant experiments has been attributed to the existence of an ‘ecological memory’ depending on the natural feeding conditions experienced by individuals previous to any experiment [9,10,11,33,34]. However, the fact that some physiological and morphological differences persist after a long period of maintenance in mussels of both origins suggests that part of the origin-related differences must owe to genotypic factors that influence the physiological rates [3, 5, 26].

### 4.2. Physiological basis for inter-individual growth variability

In this study we measured the feeding, digestive and metabolic physiological components that integrate the Scope for Growth in mussels from two origins (intertidal and subtidal) and two growth groups (fast ‘F’ and slow ‘S’), to clarify the physiological basis for inter-individual growth differences. In this section we discuss the differences found between the growth groups.

Inter-individual growth differences between S and F mussels reared under the same experimental conditions corresponded to physiological differences in the feeding performance (CR and IR), digestion rate (AR), excretion costs (VNH_4_-N) and consequently, the resulting Scope for Growth. The absorption efficiency (AE) and oxygen consumption (VO_2_) were similar for F and S growers.

Fast growth in bivalves is achieved by a combination of increased rates of feeding, reduced metabolic rates and lower metabolic costs of growth [35]. Faster feeding rates do not impair flexibility in feeding behavior, which compensates for changes in the food quantity and quality in the marine environment [35]. In the experiments of Tamayo et al. [2,5], growth differences recorded between S and F growingbivalvescorresponded to enhanced feeding rates and higher digestive performance of F clams, coupled to reduced metabolic costs per unit of assimilated food compared to S individuals. Regarding the feeding rates, our results reported that inter-individual differences in growth were sustained by greater energy acquisition (i.e. higher CR and IR) in F growers compared to S growing bivalves. F mussels showed around a 20% increase in the CR and IR per unit mass compared to S mussels.

Our results suggested that the differences in feeding rates could be associated with morphological variability of the gill area. The higher CR of F mussels seemed to depend upon 50% larger gill filtration surface. Similarly, Tamayo et al. [2] suggested that a larger gill area, rather than the gill efficiency, appeared to underpin the greater CR of F growers. In this regard, Tedengren et al. [26] compared the physiological energetics of *M*. *edulis*transplanted from the Baltic Sea to the North Sea. The lower CR of Baltic mussels might be due to a smaller gill area, resulting from a more elongated shape typical of Baltic mussels.

Teixeira de Sousa et al. [36] have associated reduced growth rate with chromosome deletion in the gill cells. When researching the level of somatic aneuploidy in the gill cells, these authors found that F growing clams had lower levels of aneuploidy than S bivalves, agreeing with the results of Leitão et al. [37]. These studies suggest that there might be a genetic determinant behind the reduced gill filtering capacity in slow growers, which opens a new field of research to improve the understanding of aneuploidy and growth rate in bivalves.

The higher feeding rates of F mussels lead to 25% higher rates of food absorptionper unit mass compared to S mussel seeds. Even if F mussels increased the intake of food, fast-growing individuals showed similar absorption efficiency as S mussels (mean AE = 80%). Likewise Tamayo et al. [2,4] reported higher AR in F bivalves but similar values of AE for F and S clams, meaning that fast-growing individuals are able to absorb a greater amount of food with the same efficiency as slow growers. Fig 4A shows the AR (J h^−1^) and VO_2_ (J h^−1^) against the SFG in mussels from both origins (subtidal and intertidal) and growth groups (F and S). Concurrently, we can infer that the highest SFG is associated with increased rates of absorption, although reduced costs of VO_2_ also contribute to higher SFG. In fact, F mussels absorbed 65% more energy with low metabolic energy expenditure (VNH_4_-N plus VO_2_) (Fig 4B). F oysters also processed more food than S spat at a low metabolic cost (Tamayo et al., 2014). Furthermore, metabolic costs in F clams represented 55% of the absorbed energy compared to 76% in S clams [4]. Despite the fact that the O:N index values were slightly higher for S mussels than F mussels, they were always above 30, the threshold given by Widdows [20] to indicate a situation of nutrition stress, proving that mussels were in an appropriate nutritional state after laboratory maintenance.

Given that F and S mussels had similar VO_2_ and that energy losses due to ammonia excretion are very small, the SFG reflected the differences in ingestion and absorption between F and S mussels rather than differences in metabolic expenditure. Fast growing mussels showed higher ingestion rates (30 J h^−1^) than slow growers (10.4 J h^−1^). Consequently, F growing mussels had almost 3 times higher energy available for growth and reproduction than S mussels. This increased SFG was reflected in a highergrowth ratefor shell and tissue in F growers (average 196 and 23.7 mg month^−1^, respectively) than S growers (average 42.95 and 5.37 mg month^−1^, respectively). The growth efficiency was also greater for fast growing mussels than slow growers (S3 Table). Similarly, comparison of growth rates in 3 intertidal mussel beds in Ninety Mile Beach (New Zealand) highlighted that F growing mussels had significantly higher Condition Index than S growing mussel population [38]. The ratio DW shell/DW tissuewas also highest for F growers than S growers (Table 1) and according to Fischer [39] these values were within the range in which the metabolism of the soft tissues will have the potential for providing protein compounds to be transferred to the shells.

In summary, faster growth was linked to faster rates of energy acquisition, food ingestion and absorption compared to slow growing bivalves. The differences in gill filtration surface might explain the higher food capture and ingestion of fast growers. The faster rates of feeding did not demand the consumption of more oxygen relative to that respired by slow growers and did not suppose greater metabolic expenditures that will decrease the SFG of F growers. Origin related differences revealed that intertidal mussels allocated more energy for the production of a thicker shell, while subtidal mussels directed more energy to tissue growth, eventually resulting in a higher food capture surface in the gills and higher energy acquisition.

## Supporting Information

S1 Data Microsoft Excel file with the complete data set used for the preparation of this study. (XLS)

S1 Table Two-way ANOVA testing significant differences in biometric parameters between mussel seed from two growth groups (fast and slow-growers) and two origins (subtidal and intertidal). (DOCX)

S2 Table Two-way ANOVA testing significant differences in physiological parameters between mussel seed from two growth groups (fast and slow-growers) and two origins (subtidal and intertidal). (DOCX)

S3 Table Energetic equivalents of the total energy allocated for Shell growth and Tissue growth and the Growth Efficiency in mussels at the beginning of the experiment and after 5 months of acclimation in the laboratory to select the individuals with slow and fast growth rates. The energetic equivalents were obtained from Wolowicz, and Goulletquer (1999).
(DOCX)
