# Supplementary material for: Flexibility of Physiological Traits Underlying Inter-Individual Growth Differences in Intertidal and Subtidal Mussels Mytilusgalloprovincialis
Source: PLoS One. 2016 Feb 5;11(2):e0148245. doi: 10.1371/journal.pone.0148245 (PMC4743968; doi:10.1371/journal.pone.0148245)
Supplement: S1 Table — (DOCX) [file pone.0148245.s002.docx]

**S1 Table. Two-way ANOVA testing significant differences in biometric parameters between mussel seed from two growth groups (fast and slow-growers) and two origins (subtidal and intertidal).**

| Effect | df | MS | F-value | P-value |
| --- | --- | --- | --- | --- |
| **Ash** |  |  |  |  |
| Growth group | 1 | 107.5 | 10.7 | <0.001 *** |
| Origin | 1 | 68.3 | 6.8 | <0.01 ** |
| G. group x Origin | 1 | 19.6 | 2.0 | 0.17 ns |
| Error | 22 | 10.0 |  |  |
|  |  |  |  |  |
| **Tissue dry weight** |  |  |  |  |
| Growth group | 1 | 54172.8 | 146.1 | <0.001 *** |
| Origin | 1 | 8816.9 | 23.8 | <0.001 *** |
| G. group x Origin | 1 | 2379.7 | 6.4 | <0.001 *** |
| Error | 22 | 370.8 |  |  |
|  |  |  |  |  |
| **Shell dryweight** |  |  |  |  |
| Growth group | 1 | 3589327.6 | 391.2 | <0.001 *** |
| Origin | 1 | 159415.1 | 17.4 | <0.001 *** |
| G. group x Origin | 1 | 200872.3 | 21.9 | <0.001 *** |
| Error | 22 | 9175.2 |  |  |
|  |  |  |  |  |
| **CI** |  |  |  |  |
| Growth group | 1 | 1.5 | 0.6 | 0.45 ns |
| Origin | 1 | 35.3 | 13.5 | <0.001 *** |
| G. group x Origin | 1 | 10.7 | 4.1 | 0.06 ns |
| Error | 22 | 2.6 |  |  |
|  |  |  |  |  |
| **Gill efficiency** |  |  |  |  |
| Growth group | 1 | 0.88 | 18.87 | <0.05* |
| Origin | 1 | 1.12 | 24.08 | <0.05* |
| G. group x Origin | 1 | 0.16 | 3.47 | 0.06 ns |
| Error | 62 | 0.05 |  |  |

Ns: not significant, *p<0.05, **p<0.001, ***p<0.001.
