# Supplementary material for: Flexibility of Physiological Traits Underlying Inter-Individual Growth Differences in Intertidal and Subtidal Mussels Mytilusgalloprovincialis
Source: PLoS One. 2016 Feb 5;11(2):e0148245. doi: 10.1371/journal.pone.0148245 (PMC4743968; doi:10.1371/journal.pone.0148245)
Supplement: S2 Table — (DOCX) [file pone.0148245.s003.docx]

**S2 Table. Two-way ANOVA testing significant differences in physiological parameters between mussel seed from two growth groups (fast and slow-growers) and two origins (subtidal and intertidal).**

| Effect | df | MS | F-value | P-value |
| --- | --- | --- | --- | --- |
| **CR _SPC_** |  |  |  |  |
| Growth group | 1 | 83.92 | 11.99 | <0.001*** |
| Origin | 1 | 55.00 | 7.85 | <0.001*** |
| G. group x Origin | 1 | 1.16 | 0.17 | 0.68 ns |
| Error | 65 | 7.00 |  |  |
|  |  |  |  |  |
| **OIR _SPC_** |  |  |  |  |
| Growth group | 1 | 80.82 | 20.57 | <0.001*** |
| Origin | 1 | 73.84 | 18.80 | <0.001*** |
| G. group x Origin | 1 | 0.07 | 0.02 | 0.89 ns |
| Error | 65 | 3.93 |  |  |
|  |  |  |  |  |
| **AR _SPC_** |  |  |  |  |
| Growth group | 1 | 33.70 | 20.18 | <0.001*** |
| Origin | 1 | 22.59 | 13.52 | <0.001*** |
| G. group x Origin | 1 | 1.75 | 1.05 | 0.31 ns |
| Error | 65 | 1.67 |  |  |
|  |  |  |  |  |
| **AE** |  |  |  |  |
| Growth group | 1 | 0.26 | 0.01 | 0.90 ns |
| Origin | 1 | 4.66 | 0.25 | 0.62 ns |
| G. group x Origin | 1 | 51.96 | 2.77 | 0.10 ns |
| Error | 37 | 18.79 |  |  |
|  |  |  |  |  |
| **VNH4-N _SPC_** |  |  |  |  |
| Growth group | 1 | 3126.22 | 56.35 | <0.01** |
| Origin | 1 | 21782.58 | 392.64 | <0.001*** |
| G. group x Origin | 1 | 111.89 | 2.01 | 0.16 ns |
| Error | 65 | 55.47 |  |  |
|  |  |  |  |  |
| **VO2 _SPC_** |  |  |  |  |
| Growth group | 1 | 0.01 | 0.36 | 0.54 ns |
| Origin | 1 | 1.83 | 40.37 | <0.001 *** |
| G. group x Origin | 1 | 0.09 | 2.01 | 0.16 |
| Error | 65 | 0.04 |  |  |
|  |  |  |  |  |
| **SFG** |  |  |  |  |
| Growth group | 1 | 2933.31 | 197.91 | <0.001 *** |
| Origin | 1 | 14.05 | 0.95 | 0.33 ns |
| G. group x Origin | 1 | 14.89 | 1.00 | 0.31 ns |
| Error | 65 | 14.82 |  |  |

Ns: not significant, **p<0.001, ***p<0.001.
