# Supplementary material for: Flexibility of Physiological Traits Underlying Inter-Individual Growth Differences in Intertidal and Subtidal Mussels Mytilusgalloprovincialis
Source: PLoS One. 2016 Feb 5;11(2):e0148245. doi: 10.1371/journal.pone.0148245 (PMC4743968; doi:10.1371/journal.pone.0148245)
Supplement: S3 Table — The energetic equivalents were obtained from Wolowicz, and Goulletquer (1999). (DOCX) [file pone.0148245.s004.docx]

**S3 Table. Energetic equivalents of the total energy allocated for Shell growth and Tissue growth and the Growth Efficiency in mussels at the beginning of the experiment and after 5 months of acclimation in the laboratory to select the individuals with slow and fast growth rates. The energetic equivalents were obtained from Wolowicz, and Goulletquer(1999).**

| Origin | Length | OW tissue | OW shell | Energyshell | Energytissue | Total energy | Rategrowth | Rategrowth | Rategrowth | Growthefficiency |
| --- | --- | --- | --- | --- | --- | --- | --- | --- | --- | --- |
| and | (mm) | (mg) | (mg) | (joules) | (joules) | (joules) | shell | tissue | total energy | (%) |
| growthgroup |  |  |  |  |  |  | (J/month) | (J/month) | (J/month) |  |
| *Initialrate* |  |  |  |  |  |  |  |  |  |  |
| Subtidal | 19-21 | 7.52 | 14.08 | 281.6 | 179.7 | 461.3 |  |  |  |  |
|  |  |  |  | **(61.04)** | **(38.96)** |  |  |  |  |  |
| Intertidal | 19-21 | 6.84 | 22.27 | 445.4 | 163.5 | 608.9 |  |  |  |  |
|  |  |  |  | **(73.15)** | **(26.85)** |  |  |  |  |  |
|  |  |  |  |  |  |  |  |  |  |  |
| *Acclimation* |  |  |  |  |  |  |  |  |  |  |
| **Subtidal** |  |  |  |  |  |  |  |  |  |  |
| Slowgrowers | 20-23 | 16.59 | 29.42 | 588.4 | 396.5 | 984.9 | 61.4 | 43.4 | 104.7 | 1.7 |
|  |  |  |  | **(59.74)** | **(40.26)** |  | **[1.9]*** | **[1.8)*** | **[1.8]*** | ***53.8**** |
| Fastgrowers | 36-39 | 46.75 | 81.51 | 1630.2 | 1117.3 | 2747.5 | 269,.7 | 18.5 | 457.2 | 2.5 |
|  |  |  |  | **(59.33)** | **(40.67)** |  | **[1.7]**** | **[1.7]**** | **[1.7]**** | ***64.7***** |
| **Intertidal** |  |  |  |  |  |  |  |  |  |  |
| Slowgrowers | 20-23 | 12.00 | 30.52 | 610.4 | 286.8 | 897.2 | 33.0 | 24.7 | 57.7 | 0.9 |
|  |  |  |  | **(68.03)** | **(31.97)** |  |  |  |  |  |
| Fastgrowers | 36-39 | 29.70 | 62.70 | 1254.0 | 709.8 | 1963.8 | 161.7 | 109.3 | 271.0 | 1.6 |
|  |  |  |  | **(63.85)** | **(36.15)** |  |  |  |  |  |

The values in bold and in parentheses indicate the % of the total energy allocated for the shell growth or the tissue growth.

The values in bold and brackets are rates of growth for the shell and the tissue for [slow growers *] and [fast growers **]

The values in bold and italics correspond to % of growth efficiency for *slow growers** and *fast growers***
